# Unmasking the Silent Culprit: Gastric Neuroendocrine Tumor in the Setting of Iron Deficiency Anemia

**DOI:** 10.7759/cureus.86010

**Published:** 2025-06-14

**Authors:** Ashley Serjilus, Sarah K Zimmer, Allison M Bush, Javier N De Luca-Johnson, Bryan J Keenan, Amie L Harvey

**Affiliations:** 1 Internal Medicine, Naval Medical Center Portsmouth, Portsmouth, USA; 2 Gastroenterology, Naval Medical Center Portsmouth, Portsmouth, USA; 3 Pathology, Naval Medical Center Portsmouth, Portsmouth, USA; 4 Gastroenterology, Naval Hospital Jacksonville, Jacksonville, USA

**Keywords:** gastric neuroendocrine tumor, iron deficiency anemia, menorrhagia, neuroendocrine, pre-menopause

## Abstract

Gastric neuroendocrine tumors (GNETs) are uncommon neoplasms; it is classified into four subtypes based on low to high risk of progression and developing metastasis. GNETs can be asymptomatic or present with nonspecific signs and symptoms, including dyspepsia and iron deficiency anemia (IDA). In people who menstruate, iron deficiency is often attributed to menstrual cycles, and they may not undergo investigation for gastrointestinal sources. We present a case of a young female with long-standing iron deficiency and menorrhagia, who was found to have a rare GNET, most consistent with a type 3 subset.

## Introduction

Gastric neuroendocrine tumors (GNETs) are rare neoplasms arising from the neuroendocrine cells within the stomach; they occur in one to two per 1,000,000 people [1]. GNETs are classified into four subtypes of GNETs based on endoscopic and histologic appearance, which have different treatment plans and different risks of metastasis and overall prognosis. They are subclassified into four types based on clinical context, endoscopic features, and histologic characteristics. Type 1 GNETs are associated with chronic atrophic gastritis and are typically small, multiple, and have low malignant potential [1]. Type 2 tumors arise in the setting of Zollinger-Ellison syndrome and multiple endocrine neoplasia type 1 (MEN1), and carry an intermediate risk of metastasis [2]. Type 3 tumors are sporadic, usually solitary, larger, and more aggressive, with a higher likelihood of local invasion and distant spread [3]. Type 4 tumors represent poorly differentiated neuroendocrine carcinomas with high-grade features and an unfavorable prognosis [4].

While many patients with GNETs are asymptomatic, some present with nonspecific gastrointestinal complaints such as dyspepsia or iron deficiency anemia (IDA) [2]. In premenopausal women, IDA is often presumed to be secondary to menstrual blood loss. However, this assumption may delay the recognition of underlying gastrointestinal pathology. Notably, guidelines emphasize that persistent or unexplained IDA, regardless of gynecologic history, warrants evaluation for gastrointestinal causes, including through endoscopic examination. We present a case of long-standing IDA in a premenopausal woman with menorrhagia who was diagnosed with a rare gastric neuroendocrine tumor with features most consistent with a type 3 subset.

## Case presentation

A 38-year-old woman with an 11-year history of IDA and menorrhagia, refractory to both oral and intravenous iron therapy, presented to the outpatient gastroenterology clinic for evaluation of persistent anemia and intermittent scant hematochezia. She had no significant past medical history and denied the use of nonsteroidal anti-inflammatory drugs (NSAIDs) or anticoagulants. Her physical examination was unremarkable.

**Figure 1:**
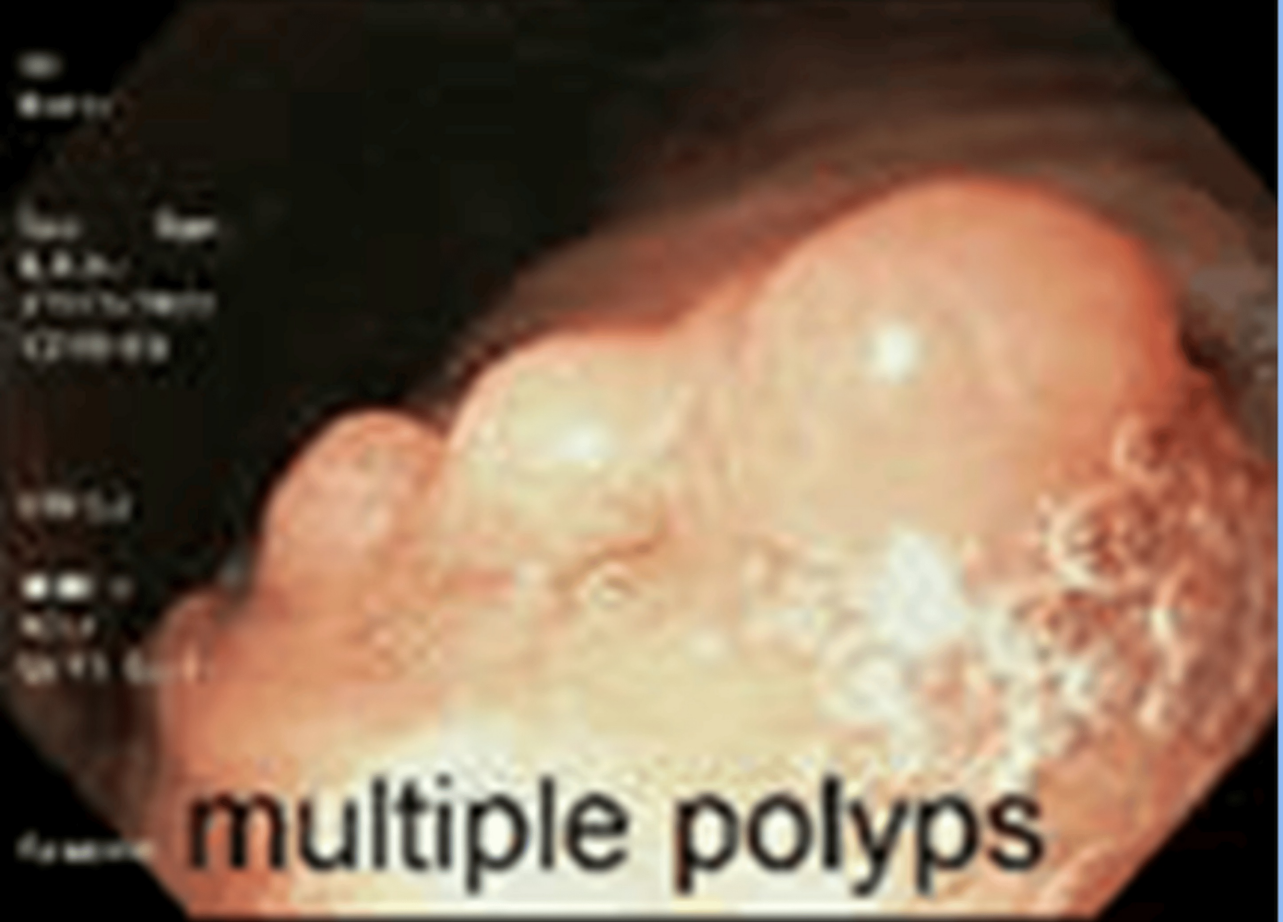
Polyps prior to polypectomy via hot snare

**Figure 2:**
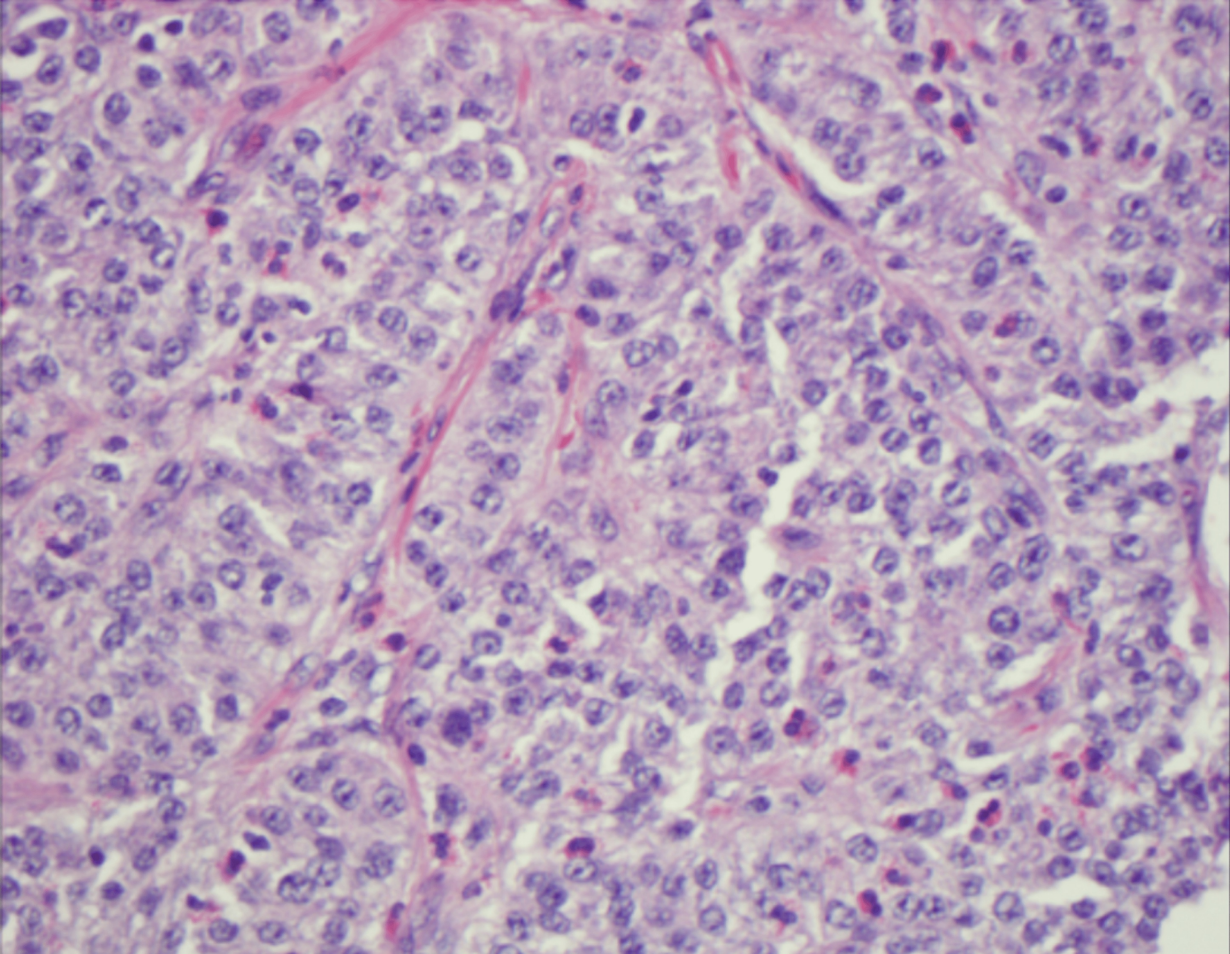
The neuroendocrine tumor demonstrates nested and trabecular architecture and in some areas is associated with a brisk chronic inflammatory response with germinal centers (H&E, high power, 400X). On high power, the neuroendocrine cells demonstrate bland cytology, with amphophilic cytoplasm, isometric nuclear contours, and finely dispersed (“salt and pepper”) chromatin with absent nucleoli. Mitotic figures are focally present and number less than two per 2 mm^2^. No lymphovascular invasion is seen

**Figure 3:**
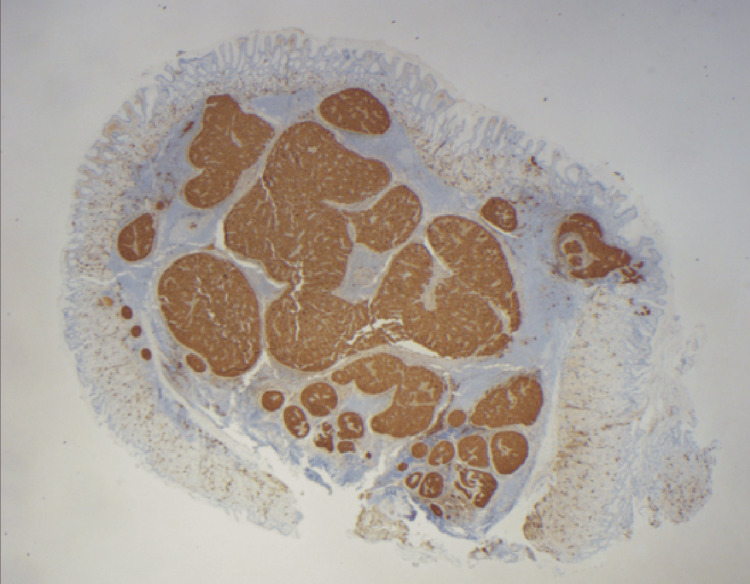
Immunohistochemical stains of neuroendocrine differentiation (synaptophysin and chromogranin) demonstrate strong and diffuse staining within tumor cells (Synaptophysin, 20X). Background gastric mucosa is negative for endocrine cell hyperplasia

**Figure 4:**
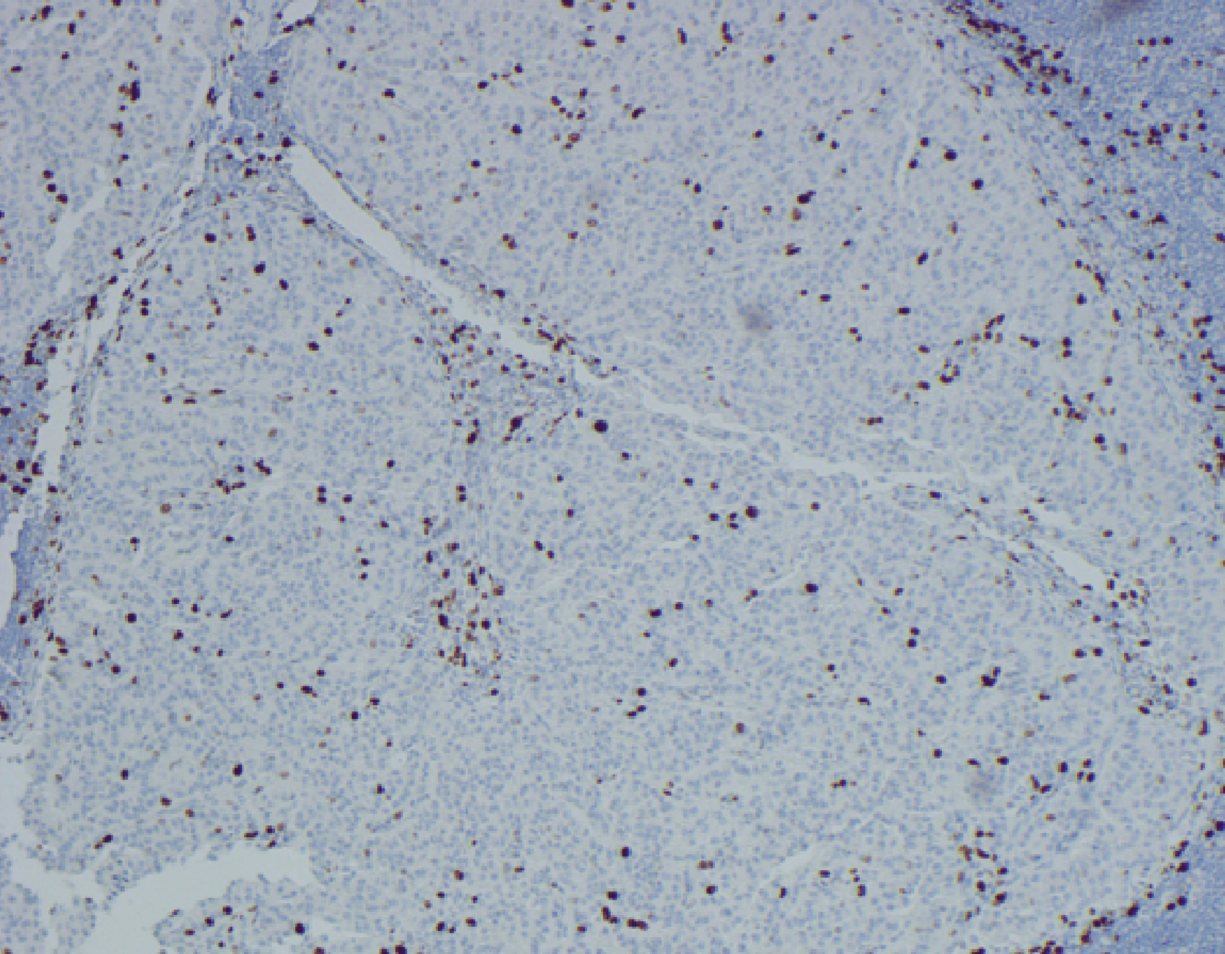
Immunohistochemical stain for Ki-67 demonstrates a labeling index of 3.2% (Ki-67, 100X); The proliferation index is determined by the manual count method utilizing camera print-out from area of highest nuclear labeling (“hot spot”). Histologic grade G2 is assigned based on the Ki-67 index

Initial laboratory studies (Table 1) revealed a hemoglobin level of 10.3 g/dL, a mean corpuscular volume (MCV) of 81 fL, a platelet count of 399 × 10^3^/μL, and a ferritin level of 29 ng/mL, findings that reflected her iron parameters following multiple courses of both oral and intravenous iron supplementation. The colonoscopy was unremarkable. Esophagogastroduodenoscopy (EGD) revealed four polyps located in the lesser curvature of the stomach, ranging from 3 to 10 mm in diameter (Figure 1). The largest polyp was resected for histopathologic analysis (Figures 2-4).

**Table 1:** Laboratory investigations MCV: mean corpuscular volume

Variables	Results	Normal range (women)
Hemoglobin, g/dL	10.3	12.0-15.5
MCV, fL	81	80-100
Platelets x 10^9^/L	399	150-400
Ferritin	29 ng/mL	15-150 ng/mL
Gastrin	22 pg/mL	13-115 pg/mL
Chromogranin A	31.6 ng/mL	19.4-98.1 ng/mL

Pathology revealed a well-differentiated neuroendocrine tumor (NET) with submucosal invasion. The tumor exhibited a Ki-67 proliferation index of 3.2% and a mitotic rate of <2 mitoses per 2 mm², classifying it as a World Health Organization (WHO) Grade 2 (G2) NET (Figure 4). Further evaluation included a gastrin level of 22 pg/mL and a chromogranin A level of 31.6 ng/mL, both within normal limits. A contrast-enhanced CT scan of the abdomen identified a subcentimeter arterially enhancing liver lesion with benign radiographic features. A PET scan showed radiotracer uptake in the proximal stomach, consistent with localized tumor activity.

On follow-up, repeat EGD revealed multiple diminutive fundic gland polyps. Biopsies showed no evidence of Helicobacter pylori infection, atrophic gastritis, intestinal metaplasia, or gastric neuroendocrine proliferation. Endoscopic ultrasound (EUS) demonstrated no perigastric lymphadenopathy and normal-appearing pancreas, biliary tree, and liver. Given the solitary, sporadic nature of the lesions, absence of hypergastrinemia, and histologic features, the gastric neuroendocrine tumor was deemed most consistent with a type 3 GNET. At the time of the submission of this report, the patient remained under active surveillance with serial EGDs.

## Discussion

NETs are a rare and heterogeneous group of neoplasms derived from neuroendocrine cells, most commonly found in the gastrointestinal tract, particularly the pancreas, small intestine, and rectum. GNETs account for less than 10% of all NETs. The classification of GNETs into four subtypes is key for understanding their pathophysiology, prognosis, and management strategies. Type 1 GNETs are commonly linked to autoimmune chronic atrophic gastritis or Helicobacter pylori infection. These tumors tend to have multiple lesions and are usually well-differentiated and indolent. Type 2 GNETs also have multiple lesions and are associated with MEN1 syndrome and Zollinger-Ellison syndrome. Both type 1 and type 2 GNETs have elevated gastrin levels and carry a generally favorable prognosis [1,2]. On the contrary, type 3 GNETs, as seen in our patient, manifest as sporadic, single, larger lesions with normal background gastric mucosa, normal gastrin levels, and an increased risk of metastasis due to a higher mitotic rate [3]. This subtype is the second most common, emphasizing its clinical relevance and the need for nuanced diagnostic approaches. Type 4 GNETs are very rare and are usually single, large lesions associated with hypergastrinemia and a background of atrophic gastritis. Type 4 gastric NETs are usually poorly differentiated with a high likelihood of metastasis [4]. 

The diagnostic journey in this case, involving several endoscopic and imaging modalities, mirrors the complexity inherent in GNET management. It highlights the importance of a multidisciplinary approach to accurately classify and treat these tumors, as advocated by Modlin et al. in 2008 [5]. Based on these features, our patient with a single lesion returning as a GNET, although less than 20 mm, has a high mitotic rate, normal gastrin and chromogranin A levels, and normal background gastric mucosa, making her subtype most consistent with type 3 GNET [6].

Clinical practice guidelines, including those from the European Neuroendocrine Tumor Society (ENETS) and the North American Neuroendocrine Tumor Society (NANETS), offer important recommendations for the management of GNETs [3,7]. Surgical resection is typically reserved for type 3 GNETs greater than 2 cm in size or the presence of local invasion or lymph node involvement. In this case, the lesion measured less than 2 cm, EUS showed no lymphadenopathy, and imaging did not reveal metastatic disease. Consequently, an endoscopic approach with active surveillance was selected under current guidelines. Specifically, ENETS recommends surveillance with EGD every 6-12 months for WHO G2 GNETs that have been completely resected and show no nodal or distant involvement [3,7]. Additionally, the recommendation to pursue bidirectional endoscopy rather than rely solely on iron supplementation in premenopausal women with IDA, especially when symptoms persist or iron therapy is ineffective, supports the approach taken in this case, emphasizing the need to evaluate both gastrointestinal and gynecologic sources of anemia [7].

Ultimately, this case highlights the importance of maintaining a broad differential for IDA and utilizing a stepwise, multidisciplinary approach to evaluate and manage rare gastric lesions. Ongoing surveillance with serial EGD is critical in this patient’s care to monitor for recurrence or progression, particularly given the intermediate-risk features of a WHO G2 type 3 GNET.

## Conclusions

This report adds a nuanced layer to the understanding of GNETs, emphasizing the significance of subtype identification and the intricate diagnostic pathway involved. While GNETs are an uncommon etiology of IDA, they should remain on the differential, particularly given their malignant nature. The dynamic and tailored approach to surveillance for our patients not only aligns with clinical guidelines but also underscores the necessity for ongoing vigilance in GNET management. This report contributes to the expanding body of knowledge on GNETs, the importance of considering occult gastrointestinal bleeding for chronic IDA, and the importance of a comprehensive, multidisciplinary approach for accurate diagnosis, classification, and management of these intriguing entities.
